# On a Substance Presenting the Chemical Reaction of Cellulose, Found in the Brain and Spinal Cord of Man
*By Rudolph Virchow. (Sept. 4, 1853.) Virchow's Archiv. B. VI. H. 1. p. 135.


**Published:** 1854-04-01

**Authors:** 


### ON A SUBSTANCE PRESENTING THE CHEMICAL REACTION OF CELLULOSE, FOUND IN THE BRAIN AND SPINAL CORD OF MAN.*

It is well known that Carl Schmidt† was the first to discover in the Ascidian the presence of a principle previously known to exist only in plants, viz. *cellulose*, and to show that it was a constituent of the animal tissue. The researches of Kölliker and Löwig,‡ of Schacht,§ and of Huxley|| have established this important fact. The occurrence of this substance, however, was limited to a comparatively very low class of the Invertebrata; and the further discovery made by Gottlieb, in *Euglena viridis*, viz., that this *infusorium* contains *paramylon*, a body isomerous with starch, also had reference only to a creature in the lowest class of the animal kingdom.¶ Nothing of the kind, on the other hand, has been known as existing in the vertebrata, and it is only since the discovery by C. Bernard—that the liver produces sugar—that we have had reason to

* By Rudolph Virchow. (Sept. 4, 1853.) Virchow's Archiv. B. VI. H. 1. p. 135.

† Zur Vergleichenden Anat. d. Wirbellos. Thiere, 1845, p. 61.

‡ Ann. d. Sci. Nat., 1846, p. 193.

§ Mull. Archiv., 1851, p. 176.

|| Quart. Journ. Micr. Sci., vol. i. p. 22. 1853.

¶ The pertinacity with which German naturalists cling to the animal nature of Euglena, we must confess, is very surprising to us, who are equally satisfied that it is as much a subject of the vegetable kingdom as the motile zoospores of any Alga, such as *Volvox, Hydrodctyon, Protococcus*, &c.

suppose that substances belonging to the *amylum* series may also have a representative.

From histological considerations, it had struck me that the umbilical cord of man presented a great resemblance in structure to the cellulose tissue of the Ascidians (Wurzb. Verh. 1851. Bd. II., p. 161, *note*), and I was only the more confirmed in this notion by Schacht's observations, so that I have since directed my researches with care to the subject. But in many instances this was in vain, as, for instance, in the *ova* of Amphibia and fishes, the remarkable vitelline plates of which I described, (Zeitsch. f. wiss. Zoologie. 1852. Bd. IV., p. 240.)

I was more fortunate when, a short time since, I directed my attention to the so-termed *corpora amylacea* of the brain, upon the precise nature of which, contrasted with the other kinds of amyloid bodies in man, I had not previously arrived at any accurate notion. (Wurzb. Verh. 1851. Bd. II., p. 51.) It was now apparent that these bodies assumed a pale-blue tinge upon the application of iodine, and upon the subsequent addition of sulphuric acid, presented the beautiful violet colour which is known as belonging to *cellulose;* and which in the present instance appears the more intense from the contrast with the surrounding yellow or brown nitrogenous substance.

I have repeated this experiment so often, and with so many precautions, that I regard the result as quite certain. Not only have I instituted comparative researches in different human bodies, and in the most various localities, but I have also noticed the action of the reagents under all possible conditions. The experiment is best made in the mode adopted by Mulder and Harting, with vegetable cellulose (*vide* Moleschott, "Physiologie des Stoffwechsels," p. 103), viz., by causing the action of diluted sulphuric acid to follow that of a watery solution of iodine. The iodine solution should not be too strong, for the observation may then be impeded by its precipitation; and, on the other hand, care must be taken that the iodine exerts due action upon the substance. Owing to the volatility of the iodine, and its great affinity for animal substances, its action is usually very unequal, so that the border of the object and not the centre may be penetrated by it; or, perhaps, of spots in close contiguity, one will contain iodine and the other not. It is, consequently, always advisable to repeat the application of the iodine several times, but to avoid the addition of too much. Upon the subsequent addition of sulphuric acid, if the action have been too powerful, the result is a perfectly opaque, red-brown colour. The most certain results are obtained if the sulphuric acid be allowed to act very slowly. In fact, I have procured the most beautiful objects in allowing a preparation covered with the glass to remain undisturbed with a drop of sulphuric acid in contact with the edge of the covering-glass for from twelve to twenty-four hours. Under these circumstances, the most beautiful light violet-blue was occasionally presented. Lastly, I would just intimate that accidental mixtures of starch or cellulose may readily happen, seeing that very light fibres or minute particles from the cloths with which the object and covering-glasses have been cleaned, may very easily be left upon them, which would afterwards exhibit the same reaction as the above.

Every precaution having been taken, the following results will be obtained:—

1. The *corpora amylacea* (Purkinje) *are chemically different from the concentric-spherical corpuscles of which the brain-sand is composed,* and with which they have hitherto usually been confounded. The organic matrix of the brain-sand granules is obviously nitrogenous: it is coloured of a deep yellow by iodine and sulphuric acid. This is true not only of the sabulous matter in the pineal gland and choroid plexuses, but also of that of the Pacchionian granulations and of the *dura mater*, as well as of the dentate plates in the spinal arachnoid. In all these parts I have, in general, nowhere obtained the blue reaction, except in a few spots in the pineal gland. It would, therefore, for the future, be convenient to restrict the name of "corpora amylacea" to the bodies containing cellulose.

2. These bodies exist, so far as I have at present found, only in the substance of the *ependyma ventriculorum* and its prolongations. In this I include especially the lining of the cerebral ventricles and the transparent substance in the spinal cord described by Kölliker, as the *substantia grisia centralis* (Mikrosk. Anat. Bd. II. 1, p. 413). With respect to the cerebral ventricles, I have already repeatedly stated, that I find them to be lined throughout with a membrane belonging to the connective tissue class, upon which rests an epithelium. This membrane contains very fine cellular elements, and a matrix sometimes of more dense, sometimes of softer consistence, and is *continued on the internal aspect without any special boundary between the nervous elements.* In the deeper layers of this membrane, and in immediate contiguity with the nerve fibres, the cellulose corpuscles are found most abundantly, and they are also especially numerous where the *ependyma* is very thick. They are consequently very abundant on the *fornix, septum lucidum,* and in the *stria cornea* in the fourth ventricle. In the spinal cord, the substance corresponding to the *ependyma* lies in the middle, in the grey substance, in the situation where the spinal canal exists in the fœtus. It there forms evidently a rudiment of the obliterated canal, such as is presented in the obliteration of the posterior cornu of the lateral ventricle, which is so frequently met with. In a transverse section of the cord, it is easily recognised as a gelatinous, somewhat resistant substance, which may be readily isolated. Its cells are much larger and more perfect than those of the cerebral *ependyma.* This *ependyma spinale* forms a continuous gelatinous filament, which extends to the *filum terminale,* and might, therefore, perhaps, be most suitably described as the *central ependymal filament.* In it the cellulose granules are also found, though, as it would seem, more abundantly in the upper than in the lower portion. In other situations I have sought for these bodies in vain, and in particular I have been unable to find them in the external cortical layer of the cerebrum, or anywhere in the interior of the cerebral substance.

3. Since, from the experiment of Cl. Bernard, who produced saccharine urine by wounding the floor of the fourth ventricle in the rabbit, there appeared to be reason to conclude that the existence of cellulose was connected with that phenomenon. I sought for it also in rabbits, but in vain: I found in that situation both in the fourth, and the third, and in the lateral ventricles, a very beautiful tessellated *epithelium* with very long vibratile cilia, but no cellulose.

4. The cellulose granules, therefore, appear to be everywhere connected with the existence of the *ependyma substance* of a certain thickness, and might perhaps be regarded as a constituent of it. They occur of excessively minute size, so that the *nuclei* of the ependyma scarcely correspond with them. Can they be formed out of the latter? The larger they are, the more distinctly laminated do they appear. But there is never any indication in them of a nitrogenous admixture, recognisable by a yellow colour. The centre only is usually of a darker blue, and consequently perhaps more dense than the cortical laminæ.

5. As to an introduction of these bodies from without, such a supposition is the less probable because a similar substance is nowhere else known. We are acquainted with a series of varieties of vegetable cellulose, but the substance now in question appears to be distinguished above all by its slight power of resistance to reagents, seeing that concentrated acids and alkalies attack it more powerfully than is usually the case with the cellulose of plants.

6. In the child I have as yet sought for it in vain, so that, like the "brain-sand," it appears to arise in a later state of development, and probably may have a certain pathological import.

Since writing the above, Professor Virchow has repeated and confirmed his observations, and ascertained in addition that similar bodies also occur in the higher nerves of sense. He found them most abundantly in the soft grey interstitial substance of the olfactory nerve, less frequently in the acoustic, although the observations of Meissner (Zeitsch. f. rat. Med., N. F., Bd. III., pp. 358, 363), would indicate a proportionately great disposition to their formation in that situation. Rokitansky appears to have seen them in the optic nerve, and from an oral communication the author has learned that Kölliker has found them in the retina.

Having already stated that the *ependyma* is continued without special limitation among the nervous elements, the author goes on to observe that it is now apparent that there is a continuous extension of the same substance in the interior of the higher nerves of sense. From a series of pathological observations, he concludes that a soft matrix referrible mainly to connective-tissue substance, everywhere pervades and connects the nervous elements in the centres, and that the *ependyma* is only a free superficial expansion of it over the nervous elements. The opinion, that the epithelium of the cerebral ventricles rests immediately upon the nervous elements, appears to have arisen from a confusion of this interstitial substance with the true nerve-substance.

The isolation of the *corpora amylacea* in larger quantity, in order that they should be subjected to chemical analysis, the author has not yet succeeded in effecting. Nevertheless it seems impossible to entertain any doubt as to their cellulose nature. No other substance is known which affords the same reaction; and although the author has examined the most various animal tissues, and has accurately investigated, particularly, the concentric corpuscles occurring elsewhere, as in the *thymus* in *tumours*, &c., nothing of the same kind has presented itself.—(*Sept.* 25, 1853.)

An abstract of the above observations also appears in the 'Comptes Rendus,' for the 26*th Sept.*, 1853, p. 492, but containing nothing additional.

Being desirous of verifying the above observations, I have examined the brains of one or two individuals; and, as my results differ in some respects from those of Professor Virchow, I will here briefly state them, leaving a more detailed account of the matter to a future opportunity, my observations at present having been too scanty to justify the expression of any settled opinion. The first case I examined was that of a young man who died of the consecutive fever of cholera, after an illness of five or six days, during the whole of which period, the renal secretion was completely suppressed. What I noticed in this case was :—

1. The enormous abundance of the *corpora amylacea* in certain situations, as the *ependyma ventriculorum*, particularly on the *septum lucidum*, and more especially also on the choroid plexuses, upon gently scraping the surface of which a fluid was obtained containing these bodies in the most surprising quantity.

2. That they existed in immense abundance in the olfactory bulbs and in the superficial parts of the brain, both cortical and medullary, contiguous to the tract of the olfactory nerves. But scarcely any part of the *cerebrum* and *cerebellum* could be examined, at all events towards the surface, without meeting with some or more; and they occurred abundantly in the very middle of the *cerebellum*. Their distribution, however, was very irregular, inasmuch as they abounded in some spots and were nearly, if not altogether wanting, in others. I could find none in the *corpora striata*, where they seemed to be replaced by "brain-sand," of which more will be said afterwards.

3. The cerebral substance in immediate contiguity with the *corpora amylacea* appeared quite natural.

4. The corpuscles were starch and not cellulose, and possessed all the structural, chemical, and optical properties of starch, as it occurs in plants, as the following few details will show :—

They were of all sizes, from less than a blood-disc up to 1-500th inch or more—generally more or less ovate, but many irregular in outline, and apparently flattened, as all the larger kinds of starch I believe are. Many of the larger ones showed the appearance which, in starch, has been erroneously described as indicative of a laminated structure; whilst in others this appearance under any mode of illumination certainly did not exist. The point that would correspond with the so-called *nucleus* of a starch-grain was, unlike that of most kinds of starch, central, and consequently the laminated marking was concentric to the grain, which is rarely the case in the starch of plants. This apparent lamination depends, as I believe, upon the same circumstances as in other starch (*vide* Trans. Micr. Soc., Quart. Journ., vol. i., p. 58), that is to say, upon the corrugation of a thin *sacculus*. That this was the case I satisfied myself by the use of sulphuric acid and of Schultz' solution (chloride of zinc and iodine), in the mode described in my paper above quoted. By these means, but more readily and conveniently by far by the latter, the *corpora amylacea* could be seen to unfold into empty, flaccid, thin-walled, blue sacculi, six to eight times larger than the original grain. Their structure thus appearing to be identical with that of starch, the identity of their chemical composition was rendered evident with equal facility. Simple watery solution of iodine coloured them deep blue, which ultimately became perfectly black and opaque. They were soluble after swelling and expanding in strong sulphuric acid, and by heat; and, moreover, they acted upon polarized light in the same way as starch does. Some of the smaller grains exhibited a distinct and sharply-defined black cross, of which the lines crossed at angles of 45° in the middle of the grain, but in the majority, there was only a single dark line in the long diameter of the grain, and which seemed always to correspond with an irregular fissure of hilus, as it might be termed, in the same direction, which was presented in a great many of the grains, and seemed to be the indication of a partial inrolling of them, as in the starch of the horse-chestnut. This longitudinal fissure was not unfrequently crossed by a shorter one at *right* angles. When the covering-glass was closely pressed, the grains were easily crushed, breaking up in radiating cracks around the margin; and sometimes, when thus compressed, a concentric annulation would become evident, which was before inapparent.

In the *corpora striata*, as I have mentioned above, I could find few or no starch-grains, but here an appearance presented itself which seems to be connected with their formation. Many particles of sabulous matter or crystalline corpuscles of the ordinary "brain-sand," were met with, all of which, instead of lying like the starch-grains, in the midst of unaltered nerve-substance, were lodged in irregular masses of what appeared a fibrinous or immature connective tissue-substance; and, in this instance, upon the addition of iodine, each mass of crystals was found to be immediately surrounded by an irregular thickness of a transparent matter, which was turned not *blue*, but a light *purplish pink* by that reagent—a substance, in fact, closely resembling in that respect the very early condition of the cellulose wall; for instance, in *Hydrodictyon*,—an immature form, as it may be termed, of cellulose.

In a second case, that of an old man—dead of chronic dysentery, and who died comatose—I found the ventricles distended with about three ounces of clear fluid. The surface of the *ependyma* throughout all the continuous cavities was studded like shagreen with minute transparent granulations, which on microscopic examination appeared finely granular and homogeneous, or sometimes faintly fibrillated. In this case there were, I think, no *corpora amylacea* in the *ependyma* (at least I found none), nor in the central substance of the brain: a few were met with in the peripheral portions, especially on the summits of the hemispheres, and still more in the much-developed Pacchionian granulations, and there commingled with *other* concentrically-laminated bodies, which formed botryoidal masses imbedded in a stroma of immature connective tissue: these bodies, which might, to distinguish them, be termed the "chalcedonic corpuscles," were rendered yellow by iodine. In this case also, I did not notice the *quasi* cellulose-deposit around the particles of "brain-sand," but in several instances I saw minute amylaceous particles (coloured blue by iodine), contained in cells which they only partially occupied.—Geo. Busk. (From No. 7 of the *Quarterly Microscopical Journal.*)

Note.—In the "Comptes Rendus," No. 23, (Dec. 5, 1853,) are some further observations on the "Animal Substance analogous to Vegetable Cellulose," by R. Virchow, in which he announces the discovery of corpuscles presenting the same reaction as the *corpora amylacea* of the brain, in the Malpighian corpuscles of diseased human spleens—in the condition termed "waxy spleen" (Wachsmilz).

---

